# Aiming to Improve Readmissions Through InteGrated Hospital Transitions (AIRTIGHT): study protocol for a randomized controlled trial

**DOI:** 10.1186/s13063-016-1725-2

**Published:** 2016-12-19

**Authors:** Andrew McWilliams, Jason Roberge, Charity G. Moore, Avery Ashby, Whitney Rossman, Stephanie Murphy, Stephannie McCall, Ryan Brown, Shannon Carpenter, Scott Rissmiller, Scott Furney

**Affiliations:** 1 Center for Outcomes Research and Evaluation, Carolinas HealthCare System, 1540 Garden Terrace, Charlotte, NC 28203 USA; 2 Carolinas Hospitalist Group, Carolinas HealthCare System, 801 East Boulevard, Charlotte, NC 28203 USA; 3 Dickson Advanced Analytics, Carolinas HealthCare System, 720 East Morehead Street, Suite 202, Charlotte, NC 28202 USA; 4 Medical Group Acute Care Division, Carolinas HealthCare System, 1000 Blythe Boulevard, Charlotte, NC 28203 USA; 5 Department Internal Medicine, Carolinas HealthCare System, 1000 Blythe Boulevard, Charlotte, NC 28203 USA

## Abstract

**Background:**

Hospital readmissions remain highly prevalent despite being the target of policies and financial penalties. Evidence comparing the effectiveness and costs of interventions to reduce readmissions is lacking, leaving healthcare systems with little guidance on how to improve quality and avoid costly penalties. Effective interventions likely need to bridge inpatient and outpatient settings, incorporate information technology, and use dedicated providers. Such complex innovations will require rigorous evaluation. The framework of quality improvement research provides an approach that both improves care locally and contributes to closing the current knowledge gaps for readmissions. In this trial, we will study a comprehensive intervention that incorporates these recommendations into an integrated practice unit, called *transition services*, with an aim of reducing 30-day readmission rates.

**Methods/design:**

We describe a nonblinded, pragmatic, controlled trial with two parallel groups comprising an evaluation of the effect of referral to a provider-led integrated practice unit, inclusive of comprehensive multidisciplinary care, dedicated paramedicine providers, and virtual visits, on 30-day readmission rates for high-risk hospitalized patients. An automated risk-scoring system will randomly generate referrals to either transition services or usual care for 1520 hospitalized patients who score as high-risk for readmission. Transition services will then engage with patients in the hospital setting using a patient navigator and provide bridging outpatient services for the 30 days following discharge. All outcome data are retrieved electronically from administrative medical records. After reapplication of inclusion and exclusion criteria at the time of hospital discharge, analyses will follow the intention-to-treat principle such that patients will be analyzed on the basis of the referral group to which they were initially randomized.

**Discussion:**

The hospital transition program under study is complex and integrates the latest recommendations for readmission reduction strategies. As healthcare systems innovate to address readmissions through such complex interventions, there is significant benefit for stakeholders to have a clear understanding of the potential reach, cost, and real-world effectiveness. The pragmatic methods described here provide a template for conducting quality improvement research that fits seamlessly into existing care delivery and improvement efforts, leading to better-informed strategic decisions and the investments necessary to transform care and value for patients.

**Trial registration:**

ClinicalTrials.gov, NCT02763202. Registered 3 March 2016 (retrospectively registered).

**Electronic supplementary material:**

The online version of this article (doi:10.1186/s13063-016-1725-2) contains supplementary material, which is available to authorized users.

## Background

Hospital readmissions are highly prevalent, costly, and associated with poor outcomes [1]. As a result, readmission reduction strategies have been an early target of policy changes and related financial penalties designed to drive quality improvement [1–5]. Beginning in 2012, the Affordable Care Act established a Hospital Readmissions Reduction Program (HRRP) authorizing Medicare to decrease payments to hospitals with excess readmission rates (https://www.medicare.gov/hospitalcompare/readmission-reduction-program.html). A recent analysis showed that, compared with the year 2007 readmission rates, the 2015 readmission rates indeed declined by an average of 2% for Medicare beneficiaries but still remain at approximately 18% for patients with conditions targeted by HRRP (myocardial infarction, pneumonia, and heart failure) [2].

For hospitals seeking guidance on improving readmissions and avoiding financial penalties, high-quality evidence comparing the effectiveness of interventions and related costs is limited and conflicting [3]. Authors of reviews have found no consistent evidence that single interventions produce significant reductions in readmission rates; however, the evidence appears promising for multifaceted interventions bridging the pre- and postdischarge periods [6, 7]. Despite these findings, the most recent large, randomized trial of a postdischarge virtual ward in Canada showed no statistically significant effect on readmission and death [8]. Given the uncertainty of effectiveness and the considerable associated costs, the next generation of multifaceted interventions should be evaluated through rigorous, pragmatic evaluations that clearly identify target populations, cost, and specific implementation factors to optimize generalizability and inform future directions [3, 9–11].

Current evidence suggests this next generation of hospital transition interventions should use analytics to focus on the patients at highest risk for a readmission while incorporating the following: (a) integration of information technology, (b) home-based interventions, (c) new types of transitional care personnel, (d) dedicated transition personnel, and (e) interventions spanning both inpatient and outpatient delivery settings [10]. Our local healthcare system adopted these recommendations into its efforts to improve readmissions by developing a comprehensive program called *transition services*. This program is based on the integrated practice unit (IPU) model that includes physician-led, team-based, integrated services focused on a specific segment of the population [12].

To properly evaluate this transition services program, we designed a randomized quality improvement trial, titled “Aiming to Improve Readmissions Through InteGrated Hospital Transitions” (AIRTIGHT), in which pragmatic methods are used to match the trial design to how the results will be used [13]. This quality improvement research approach is explicitly designed to both improve care locally and contribute to general scientific knowledge [14]. By incorporating the rigor of research methodologies such as randomization, quality improvement research seeks to generate valid and generalizable information that will guide the continuous process of improving care delivery [11, 15]. Similarly, pragmatic trials are designed to determine the effects of an intervention in the “real-world” setting, thus generating results with broad applicability [13, 16]. The Pragmatic-Explanatory Continuum Indicator Summary 2 (PRECIS-2) tool provides a framework for guiding pragmatic trial design to optimize applicability and can be applied to the design of quality improvement research studies [13]. PRECIS-2 assumes that trials have two study arms with unchanged usual care serving as the comparator and contains nine domains representing the applicability of the trial’s eventual results to real healthcare settings. This paper outlines the AIRTIGHT study protocol following both the Standard Protocol Items: Recommendations for Interventional Trials (SPIRIT) guidelines for intervention trials and the PRECIS-2 criteria for pragmatic trials [13, 17]. (The SPIRIT checklist and figure are included as Additional files 1 and 2.)

## Methods/design

### Overview

**Fig. 1:**
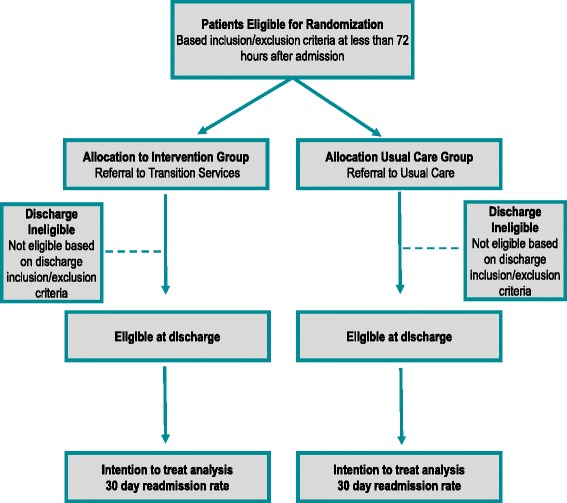
Study design and patient flow

**Fig. 2:**
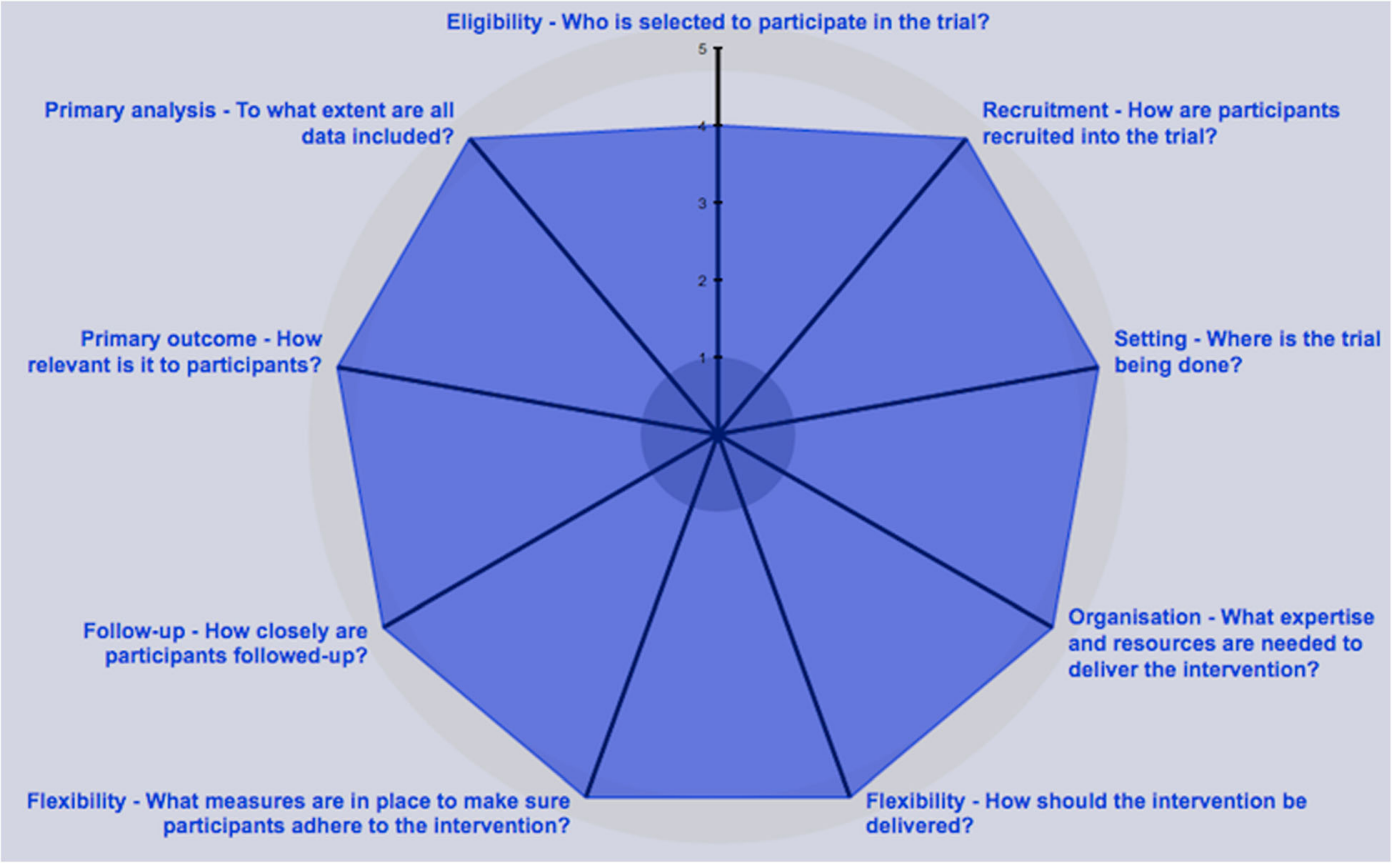
Pragmatic-Explanatory Continuum Indicator Summary 2 (PRECIS-2) tool. This framework contains nine domains representing the applicability of the trial’s eventual results to real-world healthcare settings

AIRTIGHT is a nonblinded, pragmatic, controlled trial with two parallel groups in which we are evaluating the effect of referral to a provider-led IPU, inclusive of comprehensive multidisciplinary care and virtual visits, on 30-day readmission rates for high-risk hospitalized patients (Fig. 1). The primary outcome of 30-day, all-cause, any-site readmission will be retrieved electronically from administrative medical record data. Secondary outcomes will all be retrieved in a similar fashion and include 30-day single-site readmission, emergency department use, 60- and 90-day readmissions, length of stay on index admission and readmission, and cost. To provide an understanding of implementation factors, the RE-AIM (Reach, Effectiveness, Adoption, Implementation, Maintenance) framework is incorporated into the study design [18]. The trial is pragmatic across all nine PRECIS-2 domains (Fig. 2). In keeping with this pragmatic framework, the research team has no contact with participants during any aspect of the evaluation. Randomization, patient tracking, and outcomes are all conducted electronically. The AIRTIGHT trial is registered with ClinicalTrials.gov (NCT02763202).

### Study setting

The transition services program will be evaluated in patients under the care of a hospitalist at a tertiary hospital and a smaller satellite hospital, both located within a large integrated healthcare system in Charlotte, NC, USA.

### Eligibility

Patients are eligible for referral if they are over the age of 18 years, are seen by a hospitalist, and have been categorized as an inpatient and as high-risk for readmission, defined as a risk score probability during the first 72 h of admission that is associated with a readmission rate greater than 20%. Patients are excluded if they have a primary residence more than 150 miles from the hospital; are admitted from or discharged to locations such as hospice, skilled nursing facility (SNF), or jail; leave the hospital against medical advice; have an acute psychiatric illness; have been referred as part of the AIRTIGHT evaluation in the previous 90 days; have attended a similar heart failure transitions clinic at discharge; or are being actively care managed for a cancer diagnosis or sickle cell disease. These latter groups have existing, robust care management services in place. Transition services was specifically designed to support the unique needs of patients being discharged to home; consequently, SNF patients were excluded, despite this being a group at high risk for readmission. Also, because the intervention is designed to begin during a patient’s hospital stay, the initial referrals occur early in the course of the hospitalization. However, because we expect changes in some patients’ criteria during the ensuing hospitalization course, the inclusion and exclusion criteria are applied again at discharge (Fig. 1, “Eligible at discharge” bubble).

### Risk score

The readmission risk model used is a neural network. It is built from a final set of 71 variables after the assessment of several thousand variables consisting of demographic, clinical, and use data. The model was built using retrospective information but is applied in a nearly real-time setting during a patient’s inpatient admission. Model performance was determined using a training dataset (70%) as well as a testing dataset (30%), as is typical in predictive model development. Operationally, scoring data for the model are sourced from a patient’s billing history (at the time of admission) as well as using clinical data updated throughout the hospital stay. Examples of information leveraged in the model include variables such as albumin, ammonia, body mass index, fall risk score, inability to verbalize needs, insurance type, living situation, oxygen flow rate, and pain intensity.

Every 1–4 h, a patient’s readmission risk is assessed via the model, and a risk score between 0 and 1 is calculated. This value is then attributed to one of four segments that convey increasing risk of readmission (low, moderate, high, or very high). Outside this study, clinicians use this risk information to assist in discharge planning.

### Randomization and blinding

A referral list is generated each weekday morning by randomly selecting eligible patients during their initial 72 h of admission. A six-person block randomization scheme was used so that, at any point, there are three patients assigned to usual care and three patients assigned to the transition services program. SAS software (SAS Institute, Cary, NC, USA) was used to create this 1:1 allocation, with future allocations concealed from the clinical team [19]. The total referrals are constrained to no more than 30 patients per day, but the total may be adjusted downward on the basis of daily estimates of the transition services’ capacity. Given the nature of the intervention, it is not possible to blind patients or clinicians. For the interim and final outcome analyses, a study statistician and Data and Safety Monitoring Board (DSMB) are not blinded to treatment assignment.

### Trial intervention and control

Control patients will receive usual care. Standard discharge care at both hospitals includes recommendations for follow-up appointments with a patient’s primary care physician, discharge summaries sent to primary care physicians, the arrangement of home health services based on each patient’s needs, and other outreach interventions such as follow-up care management phone calls.

In addition to usual care, patients referred to the intervention group are contacted during their hospital stay by a registered nurse patient navigator, invited to participate in the transition services program, and then followed for the remainder of their hospital stay. For 30 days following discharge, patients have access to the transition services program, which, in addition to a free-standing clinic, includes the following: (a) as-needed access to transition-dedicated internal medicine, pharmacist, paramedicine, behavioral health, and care management providers; (b) hospital follow-up evaluation with a medical provider, either virtually in the patient’s home facilitated by a paramedicine provider or in the transition clinic; (c) medication reconciliation by a pharmacist; (d) at least weekly contact with the care management team; (e) 24/7 phone support, 24/7 paramedicine visit availability, and same-day clinic scheduling; and (f) coordinated transition to the next appropriate care location after 30 days (Table 1). The intensity of the care provided is not prescribed but rather dictated by the care team and patient. At a minimum to qualify as having participated, patients will have an in-person or virtual visit with the transition services medical provider. Patients or their insurance providers are charged only for services that are billable under usual care, whereas all other services are provided by the healthcare system as part of care management.

**Table 1:** Transition services program intervention components

Transition services components	Transition recommendations [10] and drivers of readmissions addressed
Referral to transition services program and introduction by patient navigator while patient is still hospitalized	• Dedicated transition personnel • Spanning inpatient and outpatient • Engagement • Discharge plan confusion
Comprehensive postdischarge evaluation by internal medicine physician	• Dedicated transition personnel • Spanning inpatient and outpatient • Access • Timely follow-up of items outstanding at discharge • Early identification of change in patient status
Postdischarge medication reconciliation by a pharmacist	• Dedicated transition personnel • Medication errors, misunderstanding, adherence
In-home virtual appointments	• Home-based interventions • Integration of IT • Access
24/7 availability of dedicated paramedicine team for in-home visits	• Home-based interventions • New types of transitional care personnel • Dedicated transition personnel • Access • Coordinated service between home and clinic
Multidisciplinary team (internal medicine, pharmacist, paramedicine, behavioral health, and care management providers)	• Dedicated transition personnel • Access to comprehensive follow-up services
Regular care management contact starting with discharge follow-up call and weekly thereafter	• Dedicated transition personnel • Coordinated care • Engagement
Real-time population health dashboards for clinic staff	• Integration of IT
Coordinated transition to the next appropriate care location after 30 days	• Spanning inpatient and outpatient • Coordinated care • Intraprovider communication

*IT* Information technology

### Outcomes

**Fig. 3:**
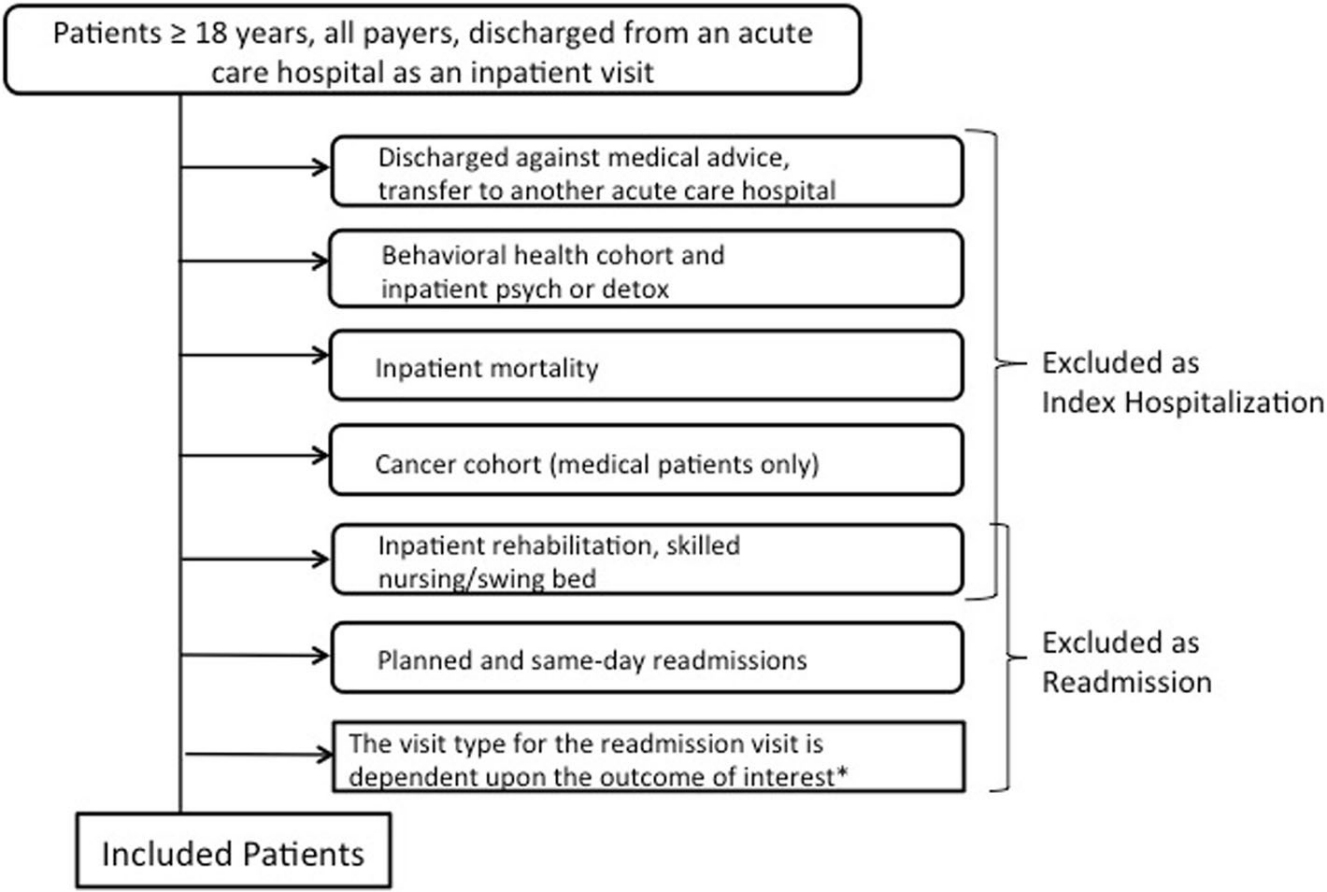
Patient-centered readmission. *The primary outcome is the difference in 30-day readmission rates between facilities. A readmission visit can be either an inpatient or an observation visit. Secondary outcomes include the difference in 30-day readmission rates between facilities. In one analysis, a readmission visit can only be an inpatient visit, and in another analysis a readmission visit can only be an observation visit

The primary outcome is the rate of 30-day, nonelective readmissions to any of the 30 hospitals affiliated with the sponsoring healthcare system. We followed the definition for nonelective readmissions as set out by the Centers for Medicare and Medicaid Services (CMS); however, we included both inpatient and observation classifications to create an outcome that is more patient-centered (Fig. 3). To allow for nondifferential outcome assessment, we use only data that are collected and available as part of routine care. Secondary outcomes include the 30-day nonelective readmission rates separated by inpatient and observation classifications (Fig. 3), 30-day emergency department use, 30-day acute care use composite (primary outcome of readmission plus emergency department use), 60- and 90-day readmission rates (using primary outcome definition of readmission), and length of stay on index and readmission visit. Additionally, in cost analysis, we will compare the total patient charges at 30, 60, and 90 days across groups with modeling of cost inclusive of estimated readmission penalties. To provide an understanding of implementation factors, RE-AIM measures are reported to the care team through monthly automated reports (Table 2) [18]. Because this evaluation is also designed to inform a quality improvement project, we expect that the clinical team may use the RE-AIM measures to adapt the transition services program during the course of the evaluation. Any substantial changes in process, procedures, or services will be documented and incorporated into the outcome analyses.

**Table 2:** RE-AIM (Reach, Effectiveness, Adoption, Implementation, Maintenance) framework

RE-AIM measure	Metrics
Reach: How do I reach the target population?	• Capture rate: proportion of patients interested in program participation out of those who are referred and appropriate at the time of discharge • Show rate: proportion of patients who have a visit with the program out of those who are referred and appropriate at the time of discharge
Effectiveness: How do I know my intervention is effective?	• Absolute and observed/expected 30-day readmission rates as defined by CMS and reported as a routine quality metric by the healthcare system
Adoption: How do I develop organization support to deliver my intervention?	• Qualitative evaluation of program and participating primary care providers, which will be reported separately
Implementation: How do I ensure the intervention is delivered properly?	• Absolute and mean visit counts and visit type by month for the clinic as a whole and per patient • Proportion of patients with a medicine reconciliation and provider visit in 72 h out of all participating patients
Maintenance: How do I incorporate the intervention so it is delivered over the long term?	• Institutional level: Reach, Effectiveness, and Implementation measures for 3 months after conclusion of trial • Patient level: 90-day readmission and composite ED and hospitalization rates • Patient level: Qualitative substudy evaluating effect of intervention more than 60 days after last contact

*CMS* Centers for Medicare and Medicaid Services, *ED* Emergency department

### Sample size

This study is designed to detect a 30% relative reduction in the readmission rate, with the usual care group assumed to have a 20% readmission rate in 30 days. A relative reduction of 30% was based on estimates from randomized controlled trials looking at bundled transition interventions and intensive home interventions [9, 20–22]. We will have 80% power to detect this reduction (transition services group rate reduced to 14%) with a total sample size of 1230 (α = 0.05) using a chi-square test for independence. Assuming 10% of those assigned to transition services will not participate, we must increase our sample size to 1520 (*n* = 760 per arm) to account for the anticipated decrease in the effect size. At the time of protocol design, available historical data included only the more narrow CMS definition of single-site readmissions (secondary outcome). Thus, the assumptions used in the power analysis are reflective of more conservative estimates than would be expected for the primary outcome measure’s expanded definition for readmission, which includes inpatient and observation stays between any of the acute care hospitals.

### Data collection

Each weekday morning, a list of eligible patients in both arms of the study is generated and stored in an SAS dataset. Patients randomized to referral to the transition services program have their information exported to an Excel file (Microsoft, Redmond, WA, USA) for use by the nurse patient navigator. It is within this file that the enrollment status is tracked by the nurse patient navigator. A patient’s choice to decline services through the transition services program is also tracked within this file. The day following list generation, enrollment status is incorporated into the SAS dataset.

Patient data such as demographics and information associated with their hospital encounters, emergency department encounters, and charges will be pulled from tables within the healthcare system’s enterprise data warehouse. Dashboards displaying patient demographics, appointment descriptions (i.e., arrived, no show, canceled visit), enrollment rates, and no-show rates are presented to the clinical teams in Tableau files [23].

### Analysis

All analyses will follow intention-to-treat principle such that patients will be analyzed on the basis of the group to which they were initially randomized, but after reapplication of inclusion and exclusion criteria at the time of hospital discharge (Fig. 1, “Eligible at discharge” bubble). Baseline comparisons of the two groups will be made using univariate analyses such as the *t* test and chi-square test. The primary outcome, readmission in 30 days, will be compared between the two groups of patients who are eligible at discharge, using logistic regression. Results will be presented with ORs and 95% CIs. In addition to the intention-to-treat analysis, we will perform a per-protocol analysis to evaluate the patients who are eligible at discharge and participate in the transition services program compared with usual care patients who are eligible at discharge. For all outcomes, we assume that if there are no visits in the electronic medical record, the value for having a visit is null; therefore, there will be no missing data for the primary or secondary outcome measures.

### Interim analysis and Data and Safety Monitoring Board

An unblinded statistician will conduct an interim efficacy analysis on the primary outcome of 30-day readmission rates once 50% of patients have been accrued in the trial and have 30-day outcomes. We will use the Haybittle-Peto procedure with a large critical value (*z* score ±3.0, two-sided α = 0.0027) [24]. Given the single planned interim analysis, no adjustment to the final critical value is needed [24]. At the same time, we will also conduct a futility analysis in which we will estimate the conditional power under the null hypothesis of no difference and the designed alternative of 30% reduction in the 30-day readmission rate. Conditional power is the probability of detecting a difference at the end of the study (*p* < 0.05), given the data trends at the time of the analysis. A recommendation will be made to stop the study for futility if the conditional power is less than 0.10. In addition to the interim efficacy and futility analyses, a sample size reestimation will also be conducted that is based on the overall readmission rate and the rate of dropouts from the intervention group, The DSMB will be composed of a senior biostatistician and three clinicians who are not affiliated with the research team. The DSMB will make recommendations to the study’s principal investigator and an executive steering committee with oversight of the evaluation.

### Ethics

The trial was approved by the Carolinas HealthCare System Institutional Review Board and granted a waiver for patient consent (reference number 01-15-10E).

## Discussion

The AIRTIGHT trial will provide important information to healthcare systems that currently have little evidence-based guidance for efforts to improve readmission rates and avoid financial penalties. First, though some trials have suggested that multifaceted interventions have potential benefit, others have suggested no benefit [6–8, 20, 22]. AIRTIGHT is the first U.S. randomized study within a large, integrated healthcare delivery system to examine the effectiveness of an intensive intervention that bridges the pre- and postdischarge periods while incorporating the most recent recommendations for hospital transitions, virtual care, and dedicated paramedicine providers. Second, the pragmatic design of this study will create information that is generalizable to other healthcare systems. We have intentionally applied the PRECIS-2 tool to match the study design to how the evidence is intended to be implemented [13]. Although randomizing patient referrals with limited inclusion and exclusion criteria increases the risk of effect-size attenuation and the study being underpowered, there is significant benefit for stakeholders to have a clear understanding of the potential reach and real-world effectiveness of the intervention. Third, the methods of the AIRTIGHT trial provide a template for healthcare systems to conduct quality improvement research that fits seamlessly into existing care delivery and quality improvement efforts. To ensure both the relevance of the questions asked and the seamless integration of the trial into clinical workflows, we formed an executive steering committee. The executive steering committee includes representation from healthcare system senior leadership and each of the major disciplines involved in the transition services program.

As the paradigm of healthcare delivery continues to evolve rapidly, the imperative increases for healthcare systems to have real-time, reliable information upon which to act. These methods should be leveraged to guide continuous improvement, strategic decisions, and investments that are necessary to transform care and value for patients.

This study has several potential limitations. First, although there are few exclusion criteria, generalizability could be threatened by the study being contained within one healthcare system. Despite this, patients are expected to be broadly representative of the general population that is at high risk for readmission because of the racially, ethnically, and geographically diverse population served by this healthcare system. Second, because we do not have access to payer-level claims data, outcomes analysis is limited to the 30 hospitals contributing clinical data to the study. If a difference in rates of hospitalization outside these 30 hospitals exists between groups, it would be expected to bias the results toward the null. Such a difference is expected because the intervention group participants should be more likely to return to the sponsoring health system’s hospitals owing to the ongoing contact with transition services personnel. Third, given the complex nature of the transition services intervention, we will not be able to isolate the effects of individual intervention components. Transition services is designed to encompass the current literature recommendations for a multifaceted intervention with the expectation that health systems would look to replicate the intervention and not its subcomponents. However, to provide an additional layer of understanding of both the intervention’s components and its implementation, a separate qualitative study will be conducted.

### Trial status

This trial is currently enrolling participants. Data collection began on 8 February 2016.

## Additional files

Additional file 1: SPIRIT 2013 checklist: recommended items to address in a clinical trial. (PDF 106 kb)
Additional file 2: Schedule of enrollment, interventions, and assessments. (PDF 75 kb)

## Abbreviations

AIRTIGHT: Aiming to Improve Readmissions Through InteGrated Hospital Transitions
CMS: Centers for Medicare and Medicaid Services
DSMB: Data and Safety Monitoring Board
ED: Emergency department
HRRP: Hospital Readmissions Reduction Program
IPU: Integrated practice unit
IT: Information technology
PRECIS-2: Pragmatic-Explanatory Continuum Indicator Summary 2
RE-AIM: Reach, Effectiveness, Adoption, Implementation, Maintenance
SNF: Skilled nursing facility
SPIRIT: Standard Protocol Items: Recommendations for Interventional Trials
