# Supplementary material for: Aiming to Improve Readmissions Through InteGrated Hospital Transitions (AIRTIGHT): study protocol for a randomized controlled trial
Source: Trials. 2016 Dec 19;17:603. doi: 10.1186/s13063-016-1725-2 (PMC5168819; doi:10.1186/s13063-016-1725-2)
Supplement: Additional file 2: — Schedule of enrollment, interventions, and assessments. (PDF 75 kb) [file 13063_2016_1725_MOESM2_ESM.pdf]

Additional File 2**.** Schedule of enrollment, interventions, and assessments

	**STUDY PERIOD**					
	**Eligibility**	**Allocation**	**Post-allocation**			
**TIMEPOINT**	$-t_1$*	**t0***	t_1**	t_2***	t_3****	t_4****
**ENROLLMENT:**						
**Initial Eligibility (<72 hours from admission)**	X					
**Allocation**		X				
**Approach and offer services**		X				
**Discharge Eligibility (*re-application of inclusion and exclusion criteria at time of discharge)***			X			
**INTERVENTIONS:**						
Transition Services program				X		
Usual care				X		
**ASSESSMENTS:**						
Demographics	X		X			
Readmission (Primary and secondary outcome definitions), ED visits, length of stay, and total charges				X		
Readmission, ED visits, and total charges					X	X

* **$-t_1$ -t0**: occurs within < 72 hours of a patient's admission to the hospital, eligibility is assessed using only data elements available in the Electronic Health Record (EHR). Allocation of referral to intervention or usual care is automated and occurs simultaneously with eligibility evaluation. Baseline demographic data are also collected at this time point.

** **t_1** : at the time of discharge inclusion and exclusion criteria are again applied using only data elements available in the EHR.

*** **t_2** : 30 day readmissions, Emergency Department (ED) visits, length of stay up to 30 days, and total charges are collected at this time point.

****$t_{3\text{-}4}$: readmissions, ED visits, and total charges are collected at 60 and 90 days after discharge from index admission.
